# Significance and Clinical Implications of Morphometric and Morphological Variability in Cadaveric Liver

**DOI:** 10.7759/cureus.59275

**Published:** 2024-04-29

**Authors:** Akanksha Deshwal, Arpita Mahajan, Shayama K Razdan, Nitin K Indora

**Affiliations:** 1 Physiotherapy, Chandigarh University, Mohali, IND; 2 Anatomy, Hamdard Institute of Medical Sciences and Research, New Delhi, IND

**Keywords:** grooves, accessory lobes, accessory fissures, quadrate lobe, caudate lobe, left lobe, right lobe

## Abstract

Background

This study aimed to identify morphological variations, conduct morphometry of the liver, and present its clinical implications.

Methodology

The study was conducted on 35 preserved cadaveric livers without macroscopic abnormalities. Morphological features such as shape and size were studied and variations such as the absence of lobe, accessory fissures, or accessory lobes were noted in all specimens.

Results

The caudate lobe was absent in one liver, and the quadrate lobe was absent in six livers. Moreover, seven livers had accessory fissures, and accessory lobes were seen in six livers. The left lobe with a lingular process was seen in three livers, and diaphragmatic grooves were present in seven livers.

Conclusions

The present research is helpful to radiologists, surgeons, and anatomists as it demonstrates the different morphological variations in the liver.

## Introduction

The liver is one of the largest organs in the abdomen. It weighs roughly 1.5 kg and has a wedge shape. It extends into the left hypochondrium and is located in the epigastrium and right hypochondrium. The function of the liver is to maintain homeostasis, providing nutrition and immune defense by performing various metabolic activities. It also helps in the breakdown of toxic substances from the blood.

The liver has five surfaces, namely, superior, inferior, anterior, posterior, and right. Its anterior and inferior surfaces are divided by a sharp inferior border. The superior surface of the liver is the largest of its five surfaces, situated underneath the diaphragm. The anterior surface has a triangular form [1].

The liver is divided anatomically into four lobes, namely, quadrate, right, left, and caudate lobes. The falciform ligament divides the right lobe from the left lobe. The left lobe is more compact than the right [2].

The caudate lobe is located on the liver’s posterior surface and is bordered on the right by the fissure for the ligamentum venosum and on the left by the groove for the inferior vena cava. It is bounded by porta hepatis below. It acts as a separate lobe and corresponds to segment 1 among the eight functional segments of the liver. The study of the morphology and variation of the caudate lobe is useful for diagnosing and analyzing clinical conditions such as liver cirrhosis [3].

The inferior surface of the liver contains the quadrate lobe. It has a fissure called ligamentum teres on the left and the gallbladder fossa on the right as its boundaries. It is confined above by the porta hepatis and below by the lower margin of the liver [1].

The liver consists of various fissures present on its posterior and inferior surfaces. Fissures for ligamentum venosum are located posteriorly between the superior portion of the left lobe and the caudate lobe, and inferiorly between the inferior portion of the left lobe and the quadrate lobe for ligamentum teres. The porta hepatis divides the quadrate and caudate lobes.

Porta hepatis is a transverse fissure that is generally 5 cm in length and is present on the inferior surface of the liver. It is present in between the caudate and quadrate lobes [4].

Significant anatomical differences have been identified through in-depth analyses of the macroscopic architecture of cadaveric livers. These differences enabled researchers to comprehend reactions to treatments used to treat liver disease. The differences found in the liver are categorized as acquired or congenital [5].

Congenital abnormalities include hypoplastic right lobe and agenesis of a hepatic lobe. Other anomalies include the absence of normal fissures, accessory lobes, and accessory fissures. Major fissures are useful in identifying liver lesions and understanding the architecture of the lobar region [6]. Linguiform lobes, a tiny left lobe in the liver, a deep renal impression, and localized liver inflammation are examples of acquired abnormalities [7].

The liver can have morphological differences such as irregularities in form, the presence of a cyst, the occurrence of one or more accessory lobes, or, less commonly, atrophy or the complete absence of a lobe.

Many studies have been done to determine the type and presence of morphological and morphometric variations in cadaveric livers. In this study, we performed some of these measurements done in previous studies to add to the existing knowledge of liver anatomy which will be useful for radiological investigations and surgical procedures on the liver.

## Materials and methods

A cross-sectional, observational study was conducted on 35 human livers from adult cadavers that were used in teaching undergraduate students. Ethical clearance was obtained from the Institutional Ethics Committee, Hamdard Institute of Medical Sciences and Research and associated HAH Centenary Hospital (approval number: EC/NEW/INST/2020/961).

A digital Vernier caliper, measuring tape, ruler, and thread were utilized for different measurements of the liver. A weighing machine was used to measure the weight of the liver.

The morphological features seen were the absence or presence of lobes, accessory fissures present on the lobes or any other surface of the liver, accessory lobes, lingular process of the left lobe, and diaphragmatic impressions seen on the anteroposterior surface of the liver. Damaged livers and livers with any surgical resection were excluded from the study.

**Figure 1:**
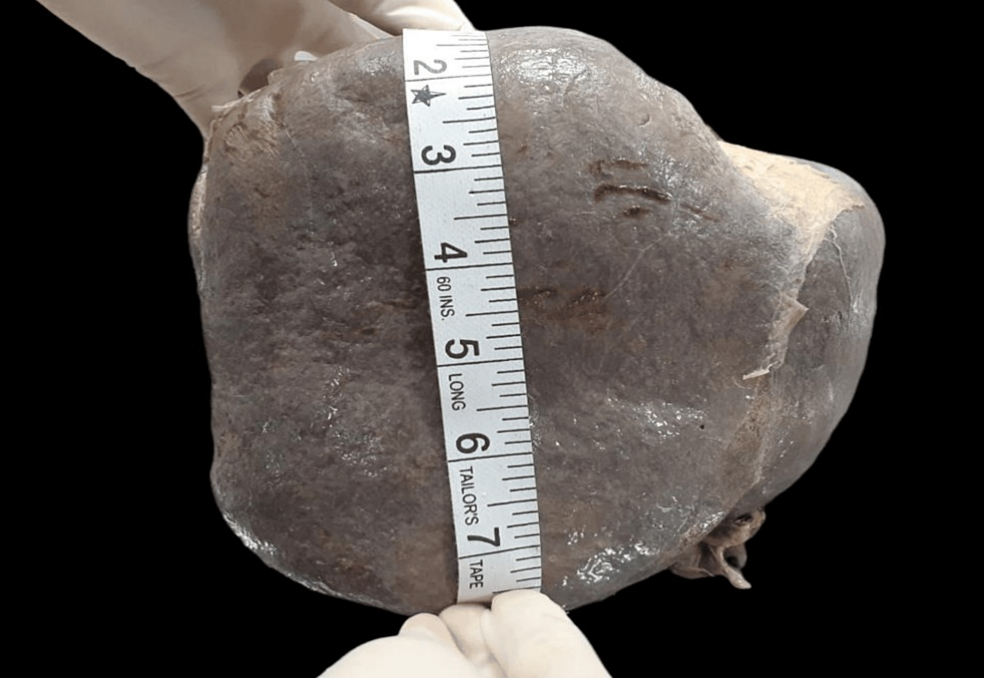
Measurement of the maximum length of the liver.

**Figure 2:**
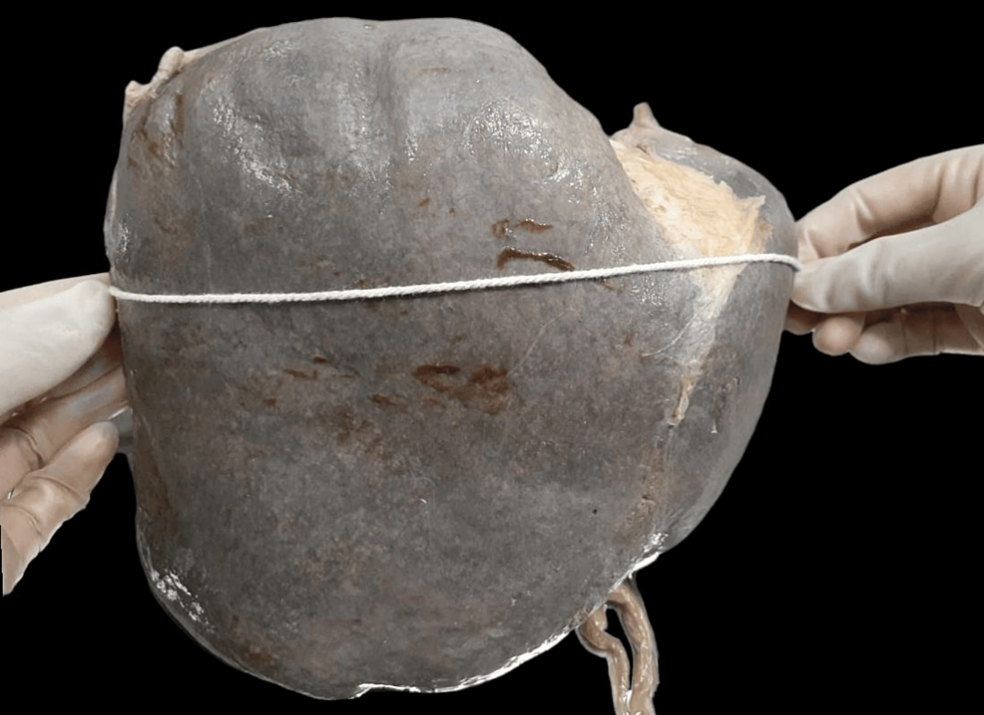
Measurement of the maximum breadth of the liver.

The liver was kept in its anatomical position to perform the morphological analysis in which the inferior vena cava was kept vertical. The maximum vertical length of the liver followed the maximum convexity of the right lobe’s upper margin to its lower margin (Figure 1). The maximum horizontal length of the liver was measured from the maximum convexity of the margin of the liver’s left lobe to the maximum convexity of the right margin of its right lobe (Figure 2).

The maximum vertical length of the left lobe was measured from its upper border to its lower border. The maximum horizontal length of the left lobe was determined by measuring from the left edge to the right edge of the left lobe. The maximum vertical length of the right lobe was measured from its upper border to its lower border. The maximum horizontal length of the right lobe was assessed from its right margin to its left margin. The length and width of the caudate and quadrate lobes were measured using a digital Vernier caliper.

## Results

A total of 35 livers were analyzed to determine the variation in morphology and morphometry. Variations observed included the presence or absence of lobes, accessory lobes, lingular process of the left lobe, accessory fissures, and grooves present on the anterosuperior surface of the liver. The livers weighed between 692.1 and 1,945.6 g. The frequencies of morphometric variations are presented in Table 1 and morphological variations of the liver are presented in Table 2.

**Table 1:** Morphometric measurements of the liver.

Measurements	Mean ± SD (cm)
Vertical length of the left lobe	13.69 ± 2.47
Horizontal length of the left lobe	7.79 ± 1.23
Vertical length of the right lobe	14.78 ± 1.84
Horizontal length of the right lobe	14.19 ± 1.93
Vertical length of the caudate lobe	5.86 ± 0.98
Horizontal length of the caudate lobe	2.68 ± 0.63
Vertical length of the quadrate lobe	6.18 ± 1.12
Horizontal length of the quadrate lobe	4.30 ± 1.19
Weight of the liver	1,292.55 ± 327.06

**Table 2:** Morphological variations of the liver.

Morphological features	N (%)
Absent quadrate lobe	6 (17.14%)
Absent caudate lobe	1 (2.85%)
Accessory lobe	6 (17.14%)
Lingular process of the left lobe	3 (8.57%)
Accessory fissure	12 (34.28%)
Diaphragmatic grooves	7 (20%)

**Figure 3:**
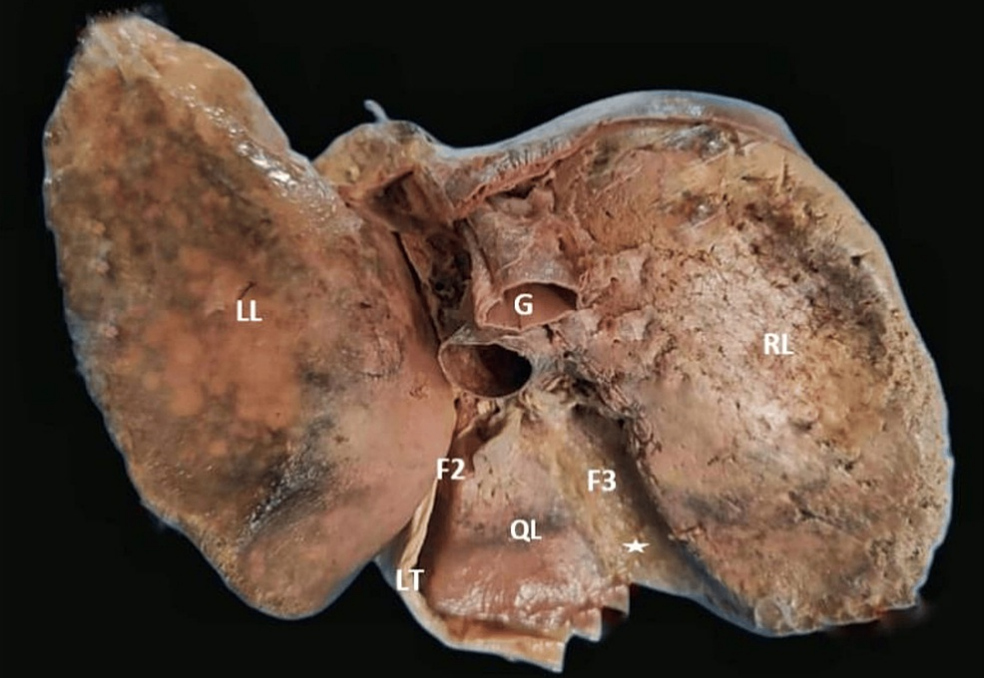
Absent caudate lobe empty gallbladder fossa. F2: fissure for ligamentum teres; F3: fossa for gallbladder (absence of gallbladder, white star); G: groove for inferior vena cava; LL: left lobe; LT: ligamentum teres; QL: quadrate lobe; RL: right lobe

**Figure 4:**
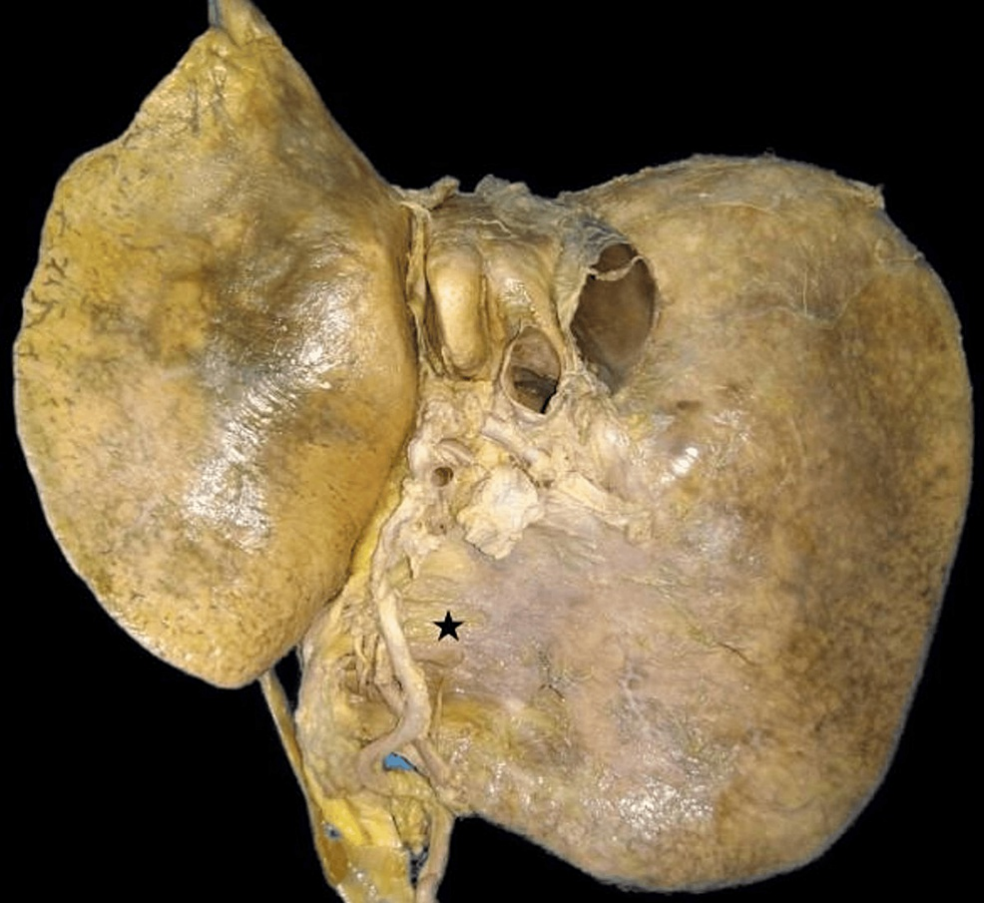
Absence of the quadrate lobe (black star) and gallbladder.

The extent of the caudate lobe, as described in the literature, was not seen in one specimen and was reported as the absence of the caudate lobe (2.85%) (Figure 3). The fossa for gallbladder did not have gallbladder, which might be due to cholecystectomy but no document was available to prove this. The quadrate lobe was absent in six (17.14%) livers (Figure 4). The right and left lobes were present in all livers.

**Figure 5:**
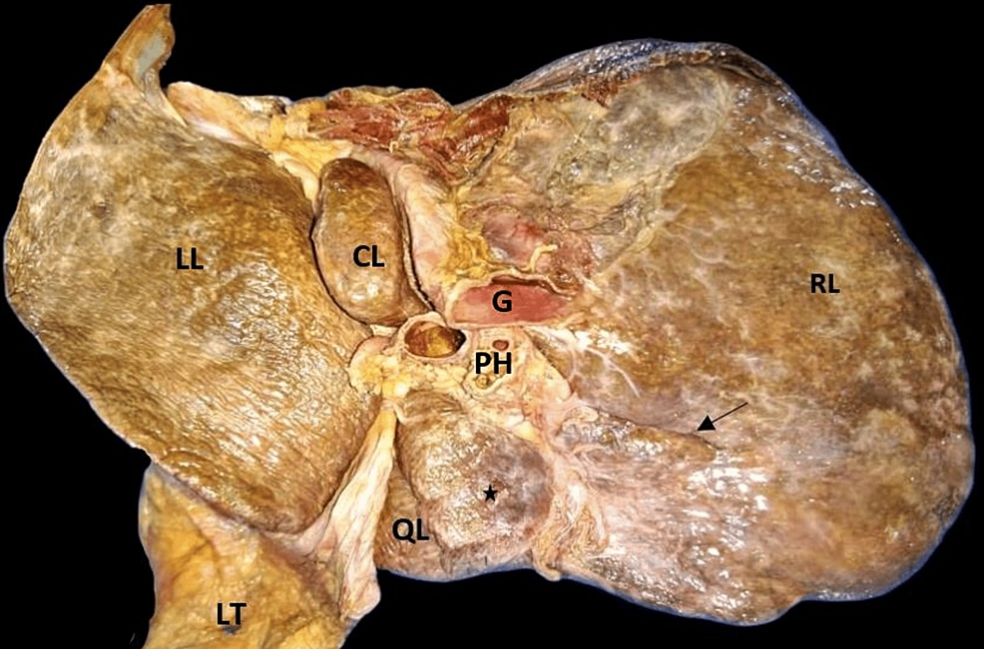
Accessory lobe (black star) and accessory fissure seen on the right lobe (black arrow). CL: caudate lobe; CP: caudate process; F1: fissure for ligamentum venosum; F2: fissure for ligamentum teres; G: groove for inferior vena cava; GB: gallbladder; LL: left lobe; LT: ligamentum teres; PH: porta hepatis; QL: quadrate lobe; RL: right lobe

Accessory lobes were found in six (17.14%) livers, among which one was found near the gallbladder along its right border which was diamond-shaped (Figure 5).

**Figure 6:**
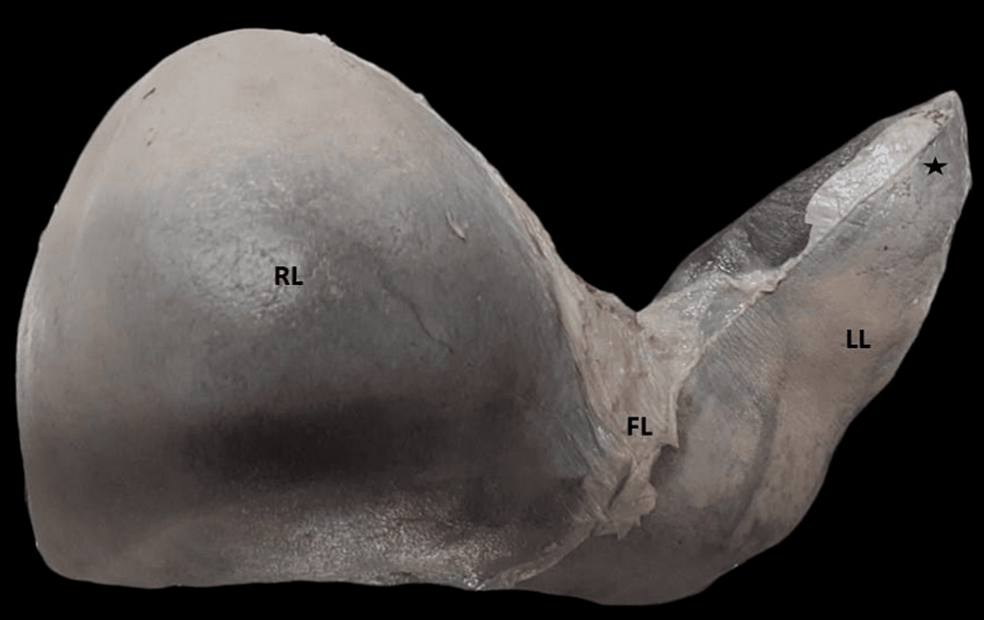
Anterior view of the liver showing the lingular process of the left lobe (black star). RL: right lobe; LL: left lobe; FL: falciform ligament

**Figure 7:**
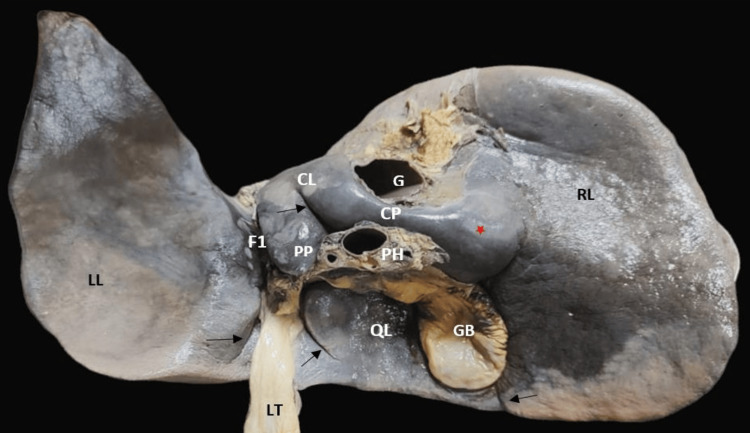
Hypertrophy of the caudate process (red star) and an accessory fissure between the papillary process and caudate process (black arrow), along with an accessory fissure on the quadrate lobe and left lobe (black arrow). CL: caudate lobe; CP: caudate process; F1: fissure for ligamentum venosum; F2: fissure for ligamentum teres; G: groove for inferior vena cava; GB: gallbladder; LL: left lobe; LT: ligamentum teres; PH: porta hepatis; QL: quadrate lobe; RL: right lobe; PP: papillary process

In total, three (8.57%) liver specimens had the lingular process of the left lobe, which is an elongation of the left lobe on its upper section (Figure 6). In two specimens, a tongue-like projection was seen over the left lobe (Figure 7).

**Figure 8:**
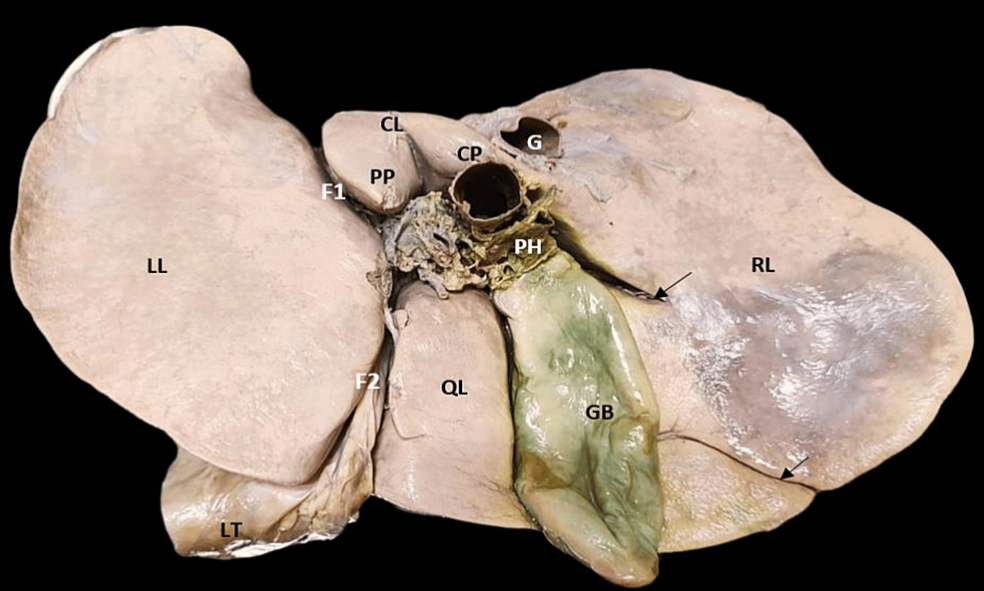
Posterior and inferior surface of the normal liver with accessory fissures (black arrow). CL: caudate lobe; CP: caudate process; F1: fissure for ligamentum venosum; F2: fissure for ligamentum teres; G: groove for inferior vena cava; GB: gallbladder; LL: left lobe; LT: ligamentum teres; PH: porta hepatis; QL: quadrate lobe; RL: right lobe

**Figure 9:**
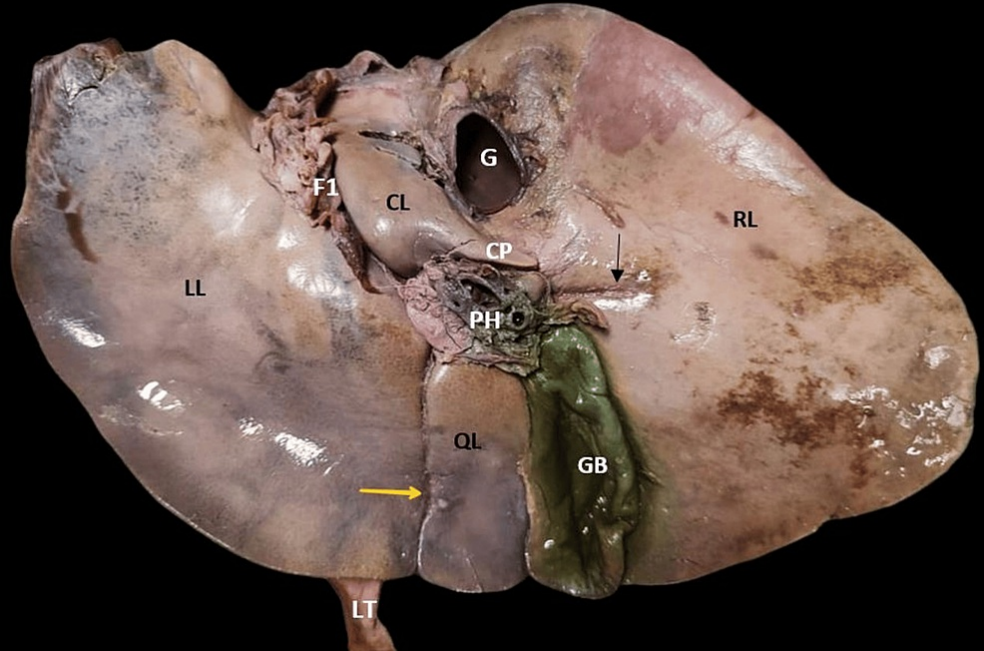
Absence of the fissure for ligamentum teres (yellow arrow) and accessory fissure near the porta hepatis on the right lobe (black arrow). CL: caudate lobe; CP: caudate process; F1: fissure for ligamentum venosum; G: groove for inferior vena cava; GB: gallbladder; LL: left lobe; LT: ligamentum teres; PH: porta hepatis; QL: quadrate lobe; RL: right lobe

Accessory fissures were noted in 12 (34.28%) livers (Figures 5, 7-9). No accessory fissures were seen on the left lobe. One specimen had an accessory fissure which was like a prominence. All fissures were obliquely placed.

**Figure 10:**
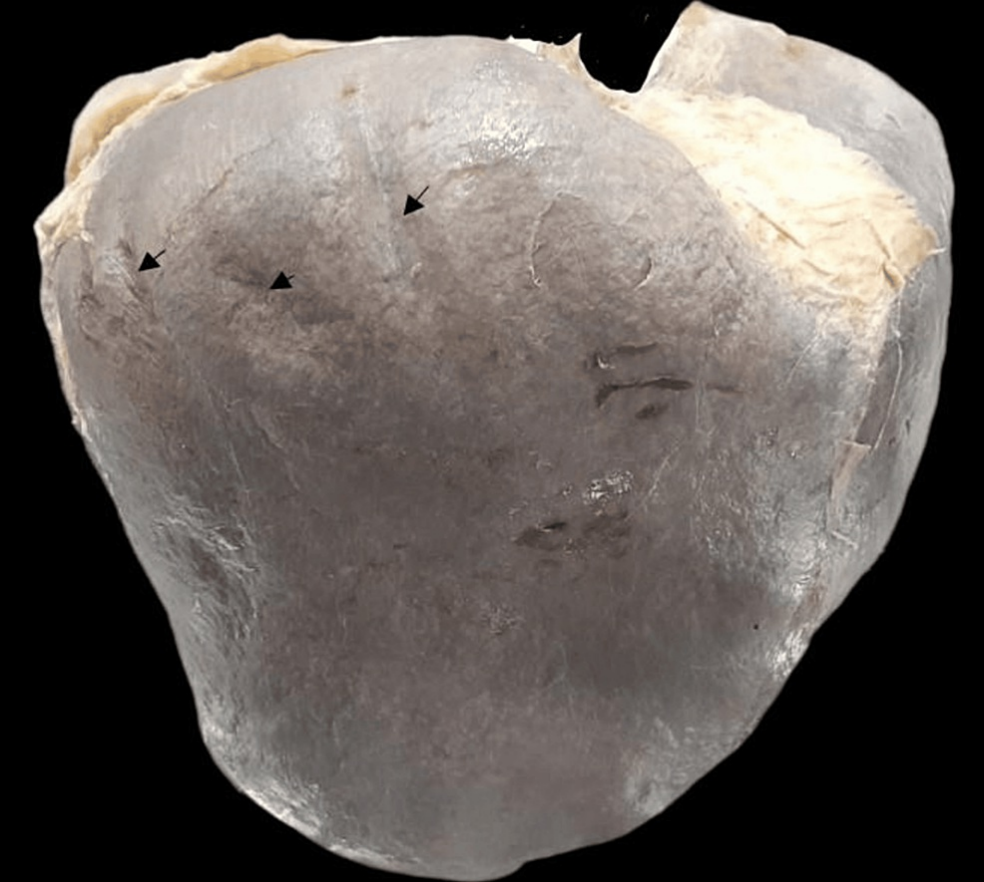
Diaphragmatic grooves on the superior surface of the right lobe of the liver (black arrows).

The liver’s anterosuperior surface was covered in impressions known as diaphragmatic grooves, which could be one or more in number (Figure 10). These grooves were found in seven (20%) livers.

One of the livers had an elevation over the quadrate lobe and one near the caudate process. Two livers had a hypertrophy of the caudate process (Figure 7).

## Discussion

The morphological features such as the external appearance, lobes, and fissures of the human liver exhibit variability. Physicians, radiologists, surgeons, and anatomists must be aware of these variations to reduce the risks associated with imaging methods and surgical procedures.

Morphological variations can be congenital or acquired. Some congenital defects include deformed lobes, agenesis of liver lobes, atrophy of the lobe, and small lobes. Many studies have investigated and reported morphological variations in the liver.

Rare occurrences are noted in variations within the right lobe of the liver. Among these, accessory fissures and accessory lobes, such as Riedel’s lobe, are the more frequently encountered variations associated with the right lobe (Figures 3, 7, 8). However, a connection between the right lobe and the quadrate lobe was found in 2.85% of the specimens in this study. In the study by Kaur et al., this was found in 15.52% of the livers [8].

Riedel’s lobe was reported by Khedekar et al. and Chaudhari et al. [9,10]. In the present study, Riedel’s lobe was not reported. Hypoplasia of the right lobe is rare and no cadaveric study has reported it yet. It was found on MRI in a study by Gathwala and Sen [11]. Alicioglu and Garg et al. found right lobe hypoplasia on MRI and CT scans [12,13]. In the present study, no hypoplastic right lobe was seen.

Left lobe hypertrophy and enlargement, commonly referred to as the lingual process, are two variations in the left lobe of the liver. In the present study, the lingular process was found in 8.57% of liver specimens, which was close to the study by Mansur et al. who noted it in 7.14% of the specimens [14]. The lingular process was reported by Anasuya et al. in 14% of livers out of 50 specimens [7]. Chaudhari et al. reported it in 12.5% of the livers [10] and Dinesh et al. in 11.4% of the cases [15]. Wahane and Satpute and Anbumani et al. found lingular process in 4% and 6.6% of livers, respectively [16,17]. The incidence was 2.7% and 1.81% in the study by Zahid et al. and Nayak, respectively [18,19]. Mamatha et al. found the maximum number of lingular processes among these studies at 21.3% [20].

Atrophy of the left lobe was reported in various studies. According to Khedekar et al., it was present in 30% of cases, while Joshi et al. reported its presence in 6.25% of the cases [9,21]. In the present study, atrophy of the left lobe was not noted.

Typically, the caudate lobe is regarded as a separate part. Enlargement of the papillary process and hypertrophy of the caudate process are two morphological variants of the caudate lobe.

In the present study, 2.8% of livers did not have a caudate lobe, which is not commonly reported in the Indian literature. However, Gardner et al. found 28.6% of livers with an absent caudate lobe in the Caribbean population [22]. Singh et al. found the division of the caudate lobe by a fissure along with a notch on its superior surface in one case [23].

The papillary process was prominent in various studies. In this study, it was found to be 17.14% which was closer to Sarala et al. who reported it in 21% of cases [24]. Hassan et al. noted it in 42.86% of livers [25] and Joshi et al. noted it in 33% of livers [2]. However, only 11.43% of livers were found with the prominent papillary process by Mansur et al. [14].

In this study, the quadrate lobe was absent in 17.14% of livers, which was similar to the study by Mansur et al. with 15.71% of cases [14]. Joshi et al. found it in 6.8% of livers [26].

Joshi et al. reported a complete absence of the fissure for the ligamentum teres in 4.5% of cases and an incomplete fissure for the ligamentum teres in 9% of cases [26]. Additionally, Simi et al. reported the absence of the fissure for the ligamentum teres in a single case [27].

The most prevalent morphological difference in the liver was the accessory lobe, caused by the liver tissue’s excessive development [10]. In this study, 17.14% of livers had an accessory lobe which was similar to the study by Saritha et al. with 16% of cases [28]. Aliya et al. found it in 10.8% of livers [18]. Mehare et al. reported it in 6% of cases [29]. Maharana and Sharma and Chaudhari et al. found it in 4.76% and 3.7% of cases, respectively [10,30].

In this study, accessory fissures were found in 34.28% of livers which was similar to the study by Mansur et al. with 32.86% and Saritha et al. with 30% of cases [14,28]. Mehare et al. found it in 27.27% of livers [29], whereas Prabahita et al. found it in 3.33% of cases [6].

Some of the studies reported percentages of accessory fissures in all lobes. Anbumani et al. found it in all the lobes, 40% in the right lobe, 3% in the left lobe, and 10% in the caudate lobe [17]. Mamatha et al. found accessory fissures in the right lobe in 10% of cases [20]. However, Saritha et al. found it in 2.5% and 2% of cases [28]. Joshi et al. found accessory fissures in the quadrate lobe among 9% of livers [26].

Several studies have reported finding grooves on the liver’s anterosuperior surface. In this study, diaphragmatic grooves were found in 20% of cases. Joshi et al. reported it in 13.6% of liver specimens [26]. Chaudhari et al. found it in 7.5% of livers [10], whereas it was found to be 4% and 2% in the study by Saritha et al. [28].

In this study, the vertical length of the right lobe ranged from 115 to 180 mm and the horizontal length from 94 to 176 mm. The left lobe had a vertical length ranging between 90 and 200 mm and a horizontal length between 60 and 100 mm. The vertical length of the caudate lobe ranged from 44 to 85 mm and the horizontal length from 14 to 36 mm. The vertical length of the quadrate lobe ranged from 38 to 76 mm whereas the horizontal length from 21 to 68 mm. These findings were compared with studies by Chavan et al., who reported a vertical length of the caudate lobe ranging between 40 and 90.3 mm and a horizontal length between 25 and 42 mm, which is closer to the present study [3]. The mean vertical length of the caudate lobe in the present study was 58.6 mm and the mean horizontal length was 26.8 mm, which is closer to the study by Gardener et al., who reported a mean vertical length of 52.6 mm and a mean horizontal length of 32.17 mm [22]. The mean horizontal length of the right lobe and the left lobe in the present study was 141.9 mm and 77.9 mm, respectively, whereas Joshi et al. reported a mean horizontal length of the right lobe of 105.6 mm and the left lobe of 64.7 mm [21].

A comparison between the findings of the various studies discussed above is presented in Table 3.

**Table 3:** Comparison of variations of morphological features with other studies.

Authors	Accessory lobe (%)	Lingular process (%)	Accessory fissures (%)	Diaphragmatic groove (%)
Nagato and Silva (2011) [5]	-	21.31	-	6.56
Baruah and Choudhury (2016) [6]	1	10	3.3	-
Geeta Anasuya et al. (2020) [7]	24	14	40	28
Mansur et al. (2019) [14]	-	7.14	32.8	-
Wahane and Satpute (2013) [16]	16	4	20	20
Nayak (2019) [19]	9.09	-	1.81	1.81
Mamatha et al. (2014) [20]	-	4	32	12
Saritha et al. (2015) [28]	16	-	30	4
Present study	17.14	8.57	34.28	20

Limitations

This study had a few limitations. The study had a small sample size. Moreover, we were unable to conduct a study taking gender into consideration.

## Conclusions

The current study conducted on 35 livers in an adult population irrespective of sex emphasizes the morphological changes in the liver, such as the diaphragmatic grooves, lingular process of the left lobe, accessory fissures, and the presence or absence of lobes. The study has recorded a variety of morphometric and morphological changes in the liver, information that anatomists would find useful during routine dissection. It is important to be aware of these variations for better diagnosis and clinical correlations in liver diseases. This variation would help radiologists for better interpretation of radiological images. Moreover, it will be useful for surgeons to reduce morbidity during surgeries.
